# Supplementary material for: REDD1 Deletion Suppresses NF-κB Signaling in Cardiomyocytes and Prevents Deficits in Cardiac Function in Diabetic Mice
Source: Int J Mol Sci. 2024 Jun 12;25(12):6461. doi: 10.3390/ijms25126461 (PMC11204184; doi:10.3390/ijms25126461)
Supplement: Supplementary file 1 [file ijms-25-06461-s001.zip › ijms-3040063-supplementary.pdf]

Supplementary Tables

Table S1. Oligonucleotides used for PCR analysis

Human Primers

Gene	Orientation	Primer Sequence (5′-3′)
Actin	F	ACCATGGATGATGATATCGCC
	R	GCCTTGCACATGCCGG
CCL2	F	CATGAAAGTCTCTGCCGCCC
	R	GGGCATTGATTGCATCTGGCTG
IL-1β	F	TTCGAGGCACAAGGCACAA
	R	TGGCTGCTTCAGACACTTGAG
IL6	F	CCCACCGGGAACGAAAGA
	R	TGGACCGAAGGCGCTTGT
REDD1	F	TGGTGCCCACCTTCCAGCTG
	R	GTCAGGGACTGGCTGAAGCC

Mouse Primers

Gene	Orientation	Primer Sequence (5′-3′)
Actin	F	ACGGCCAGGTCATCACTATTG
	R	TGGAAAAGAGCCTCAGGGC
CCL2	F	CACTCACCTGCTGCTACTCA
	R	GCTTGGTGACAAAAACTACAGC
REDD1	F	GGGATCGTTTCTCGTCCTCC
	R	ATGAGGAGTCTTCCTCCGGC

Table S2. Antibodies used for Western Blotting

Primary Antibodies

**Cell Signaling**	**Cat #**	**Lot #**	**Dilution**
GSK3β	5676S	4	1:1000
p-IKKα/β S179/180	2697S	21	1:1000
IKKβ	2678	2	1:1000
p-p65 NFκB S536	3033S	19	1:1000
p65 NFκB	8242S	16	1:1000
**Protein Tech**	**Cat #**	**Lot #**	**Dilution**
REDD1	10638-1-AP	95508	1:500
**Santa Cruz**	**Cat #**	**Lot #**	**Dilution**
α-tubulin	sc-32293	C0112	1:1000

Secondary Antibodies

**Bethyl**	**Cat #**	**Lot #**	**Dilution**
Mouse	A90-116P	43	1:10000 in TBS-T, 5% milk
Rabbit	A120-101P	44	1:10000 in TBS-T, 5% milk

Table S3. Antibodies used for Immunofluorescence

Primary Antibodies

Cell Signaling	Cat #	Lot #	Dilution
p-GSK3β S9	5558P	5	1:500
IL-1β	12242S	4	1:200
**Novus**	**Cat #**	**Lot #**	**Dilution**
CCL2	NBP1-07035AF532	D135527	1:200

Secondary Antibodies

Jackson ImmunoResearch	Cat #	Lot #	Dilution
Donkey anti-Mouse Alexafluor 488	711-545-152	164289	1:800
Donkey anti-Rabbit Alexafluor 488	715-546-151	125414	1:800
